# Redox-Mediated Gold Nanoparticles with Glucose Oxidase and Egg White Proteins for Printed Biosensors and Biofuel Cells

**DOI:** 10.3390/ijms24054657

**Published:** 2023-02-28

**Authors:** Natcha Rasitanon, Kornautchaya Veenuttranon, Hnin Thandar Lwin, Kanyawee Kaewpradub, Tonghathai Phairatana, Itthipon Jeerapan

**Affiliations:** 1 Center of Excellence for Trace Analysis and Biosensor, Prince of Songkla University, Hat Yai 90110, Thailand; 2 Division of Physical Science, Faculty of Science, Prince of Songkla University, Hat Yai 90110, Thailand; 3 Department of Biomedical Sciences and Biomedical Engineering, Faculty of Medicine, Prince of Songkla University, Hat Yai 90110, Thailand; 4 Institute of Biomedical Engineering, Faculty of Medicine, Prince of Songkla University, Hat Yai 90110, Thailand; 5 Center of Excellence for Innovation in Chemistry, Faculty of Science, Prince of Songkla University, Hat Yai 90110, Thailand

**Keywords:** glucose oxidase, gold nanoparticles, egg white proteins, biosensors, biofuel cells, self-powered biosensors, glucose, naphthoquinone

## Abstract

Glucose oxidase (GOx)-based electrodes are important for bioelectronics, such as glucose sensors. It is challenging to effectively link GOx with nanomaterial-modified electrodes while preserving enzyme activity in a biocompatible environment. To date, no reports have used biocompatible food-based materials, such as egg white proteins, combined with GOx, redox molecules, and nanoparticles to create the biorecognition layer for biosensors and biofuel cells. This article demonstrates the interface of GOx integrated with egg white proteins on a 5 nm gold nanoparticle (AuNP) functionalized with a 1,4-naphthoquinone (NQ) and conjugated with a screen-printed flexible conductive carbon nanotube (CNT)-modified electrode. Egg white proteins containing ovalbumin can form three-dimensional scaffolds to accommodate immobilized enzymes and adjust the analytical performance. The structure of this biointerface prevents the escape of enzymes and provides a suitable microenvironment for the effective reaction. The bioelectrode’s performance and kinetics were evaluated. Using redox-mediated molecules with the AuNPs and the three-dimensional matrix made of egg white proteins improves the transfer of electrons between the electrode and the redox center. By engineering the layer of egg white proteins on the GOx-NQ-AuNPs-mediated CNT-functionalized electrodes, we can modulate analytical performances such as sensitivity and linear range. The bioelectrodes demonstrate high sensitivity and can prolong the stability by more than 85% after 6 h of continuous operation. The use of food-based proteins with redox molecule-modified AuNPs and printed electrodes demonstrates advantages for biosensors and energy devices due to their small size, large surface area, and ease of modification. This concept holds a promise for creating biocompatible electrodes for biosensors and self-sustaining energy devices.

## 1. Introduction

Glucose oxidase (GOx, Enzyme Commission number: 1.1.3.4) is a flavoenzyme that catalyzes the oxidation of glucose. It is widely employed for glucose biosensors to determine the amount of glucose in body fluids. GOx has a homodimeric protein structure with an active site located in a deep pocket where glucose binding takes place [1]. The structure of GOx creates one of the most challenging tasks in biosensor development when dealing with the immobilization of GOx on an electrode surface. A variety of nanomaterials introduced for surface modification in biosensor applications have been reported, such as carbon nanotubes, nanofibers, and particularly metallic nanoparticles [2]. Gold nanoparticles (AuNPs) serve as an electrical wire between the redox-active center of the enzyme and the electrode, allowing intimate interaction with GOx [3]. This example was carried out by reconstituting apo-glucose oxidase on a 1.4-nanometer gold nanocrystal functionalized with flavin adenine dinucleotide (FAD) and incorporated into a conductive film generates a bioelectrocatalytic system with electrical contact with the electrode support. The reconstitution of GOx on AuNPs via FAD provides a high electron transfer rate that is seven times higher than that of the natural cosubstrate of the enzyme, offering excellent analytical performances of electrochemical behaviors [3]. Although AuNPs have been explored as a conducting material to receive electrons from GOx, which can improve the efficiency of electron transfer, there are still limitations to the use of DET [4]. Researchers continue to face critical challenges in immobilizing GOx on solid electrodes.

Although proteins have been applied for creating bio-matrix to host enzymes, few reports have used highly biocompatible food-based biomolecules to hold enzymes on nanoparticle-based bioelectrodes. Previously, proteins, such as bovine serum albumin (BSA) and silk fibroin, could be used as an immobilization matrix for biosensor development due to their biocompatibility, biodegradability, presence of functional groups, as well as high stability. BSA is often used in the cross-linked matrix to protect the activity of oxidase-based electrodes while enhancing the sensor stability, such as the co-immobilization of methylene green and horseradish peroxidase in the montmorillonite-modified BSA-glutaraldehyde matrix for hydrogen peroxide detection [5]. Burmeister et al. reported the use of BSA and glutaraldehyde cross-linked glutamate oxidase for glutamate analysis [6]. Another example incorporated BSA with glycerol and poly(ethylene glycol) diglycidyl ether matrix to provide the stability of glucose and lactate sensors [7]. Similarly, silk fibroin is a natural protein that possesses environmentally friendly, excellent tensile strength, and many amino groups. The molecular structure provides multiple functional groups such as −OH, −NH_2_, and −COOH, allowing the entrapment of the biomolecules without the use of any chemical reagents [8]. Along with that, it has been reported that the use of silk fibroin as supporting materials offers long-term operational stability, maintaining GOx activities to minimize the leakage of the immobilized GOx from the matrix [9,10,11]. Kuzuhara et al. introduced a colorimetric study of GOx entrapped in silk fibroin. The results showed that the silk fibroin-based biosensor could preserve the enzyme activity (98.7%) and avoid leakage of the enzymes (0.05%) after 36 days [11]. Liu and colleagues recently reported on the coupling of an enzymatic silk fibroin nanofibrils membrane via glutaraldehyde with ultrathin platinum nanoparticles/graphene film for glucose and lactate sensing. The glutaraldehyde can be crosslinked with a hydroxyl group of silk fibroin and an oxidase enzyme, thus creating a porous enzymatic nanofiber membrane or three-dimensional (3D) scaffold structure. The results revealed that the fabricated sensors provided high sensitivity and long-term stability for several hours (up to 25 and 23.6 h for glucose and lactate sensors) when retaining the enzymes in the 3D space [9]. To date, there are no reports on the use of cheap and biocompatible food-based materials, such as egg white proteins, conjugated with GOx and nanoparticles to fabricate bioelectrodes for biosensors and biofuel cells (BFCs). Similar to previously reported proteins, it is expected that a 3D network can also be formed due to the availability of amino acids in egg white proteins for the glutaraldehyde crosslinking process.

The use of glucose biosensors, self-powered glucose sensors, and BFCs can revolutionize the technology for managing diabetes mellitus, a condition characterized by high blood glucose levels because the body fails to produce enough insulin. With diabetes’ increasing prevalence and complications, glucose monitoring devices are in demand. Even though glucose sensors have been developed for many years, strategies for detecting glucose still need to be developed. Electrochemical analysis is the most widely used method for measuring glucose, thanks to its simplicity, quantitative nature, and wide detection range. As soon as the sample has been analyzed, the electrochemical signals can be converted directly into glucose concentrations [12]. These days, with the proliferation of smartphones and the growing need for real-time and continuous monitoring, wearable devices are expected to become the mainstream for glucose monitoring. Interestingly, it is possible to connect glucose levels in perspiration, saliva, urine, tears, and interstitial fluid with blood glucose levels; thus, non-invasive or minimally invasive glucose monitoring is possible [13]. With the ability to continuously measure glucose, wearable sensing has opened promising avenues for delivering information to aid in diabetes diagnosis and enable early treatment intervention. Considerable efforts have been made in the development of wearable noninvasive glucose biosensors, including sweat-based devices [14,15,16,17], tear-based devices [18,19,20,21], oral cavity-based devices [22,23,24], and interstitial fluid-based devices [25,26]. Furthermore, glucose BFCs were discovered to be useful for powering sensors, as they harvest energy from the biofluid and convert it into electricity, providing self-sustaining power sources that can be utilized to monitor their environments [27]. Recently, numerous efforts have been made to glucose-based BFC in living organisms and humans with the expectation of possible future biomedical applications and self-sustaining power generation, such as a glucose-based BFC implanted in a snail to generate electrical power [28], a glucose-based BFC in a lobster capable of powering a pacemaker and an electronic watch [29,30], and a glucose-based BFC in a pigeon to generate power for intermittent neurostimulation [31].

For safety and to avoid negative reactions, bioelectrodes should be compatible with tissue, especially for flexible wearable devices. Additionally, enhancing the stability of enzymatic electrodes is necessary for consistent monitoring and to maintain the device’s performance over time, ensuring it can function for prolonged periods. Therefore, it is essential to exploit new biocompatible molecules to create an immobilization matrix for developing bioelectrodes. We aim to design glucose biosensors and self-powered sensors, while BFCs provide a sustainable energy source for biodevices.

This work describes the first example investigating bioelectrochemical interface of GOx and egg white proteins on 5 nm AuNPs functionalized with a redox mediator molecule (1,4-naphthoquinone, NQ) and integrated on a screen-printed conductive carbon nanotube (CNT)-modified material. The effective conjugation of redox-mediated AuNPs with GOx and egg white proteins was investigated by evaluating bioelectrochemical kinetics. Different electrode configurations were studied. This bioelectrode could be applied to create an energy-harvesting device that can convert glucose into electricity. The generated electricity could also drive the self-powered biosensing module. Applying biocompatible food-based proteins and a newly engineered bioelectrode interface could improve biosensing functions and bioenergy conversion systems by connecting the GOx with redox molecule-modified nanoparticles and maximizing electron flow from the redox center of GOx for advanced biosensing applications while maintaining the activity of the GOx.

## 2. Results and Discussion

### 2.1. The Concept of Redox-Mediated AuNPs with GOx and Egg White Proteins Conjugated with a Printed CNT-Modified Amperometric Biosensor and a BFC

**Figure 1:**
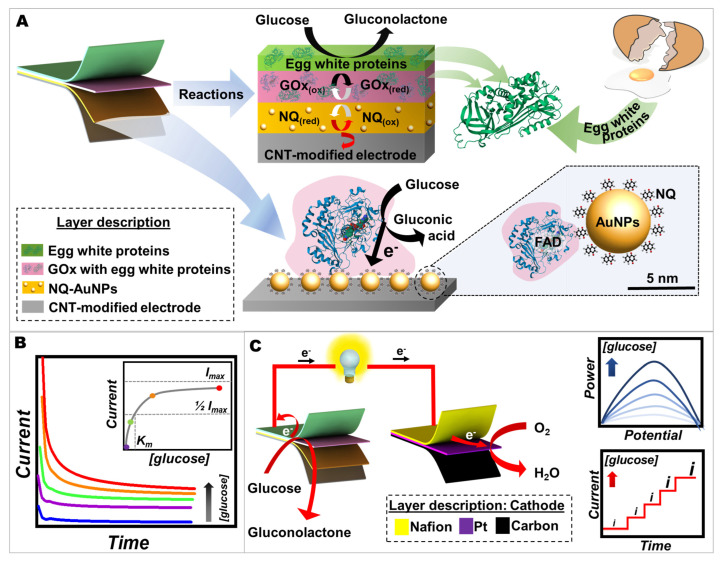
The conceptual presentation of redox-mediated AuNPs with GOx and egg white proteins for biosensors and BFCs. (**A**) The components of a screen-printed bioanode along with the redox reactions occurring on its surface. The bottom-right image represents a 5 nm AuNP functionalized with NQ and connected to GOx, drawn to scale, highlighting the size and functionalization of the spherical particle. (**B**) Amperometric responses of the screen-printed GOx-based electrode to successive additions of glucose solution. The inset shows the corresponding Michaelis–Menten plot. (**C**) The components of a screen-printed glucose BFC and its reactions along with the operation for energy-harvesting and self-powered sensing modules.

We describe a new electrochemical biosensing interface relying on GOx with egg white proteins and 5 nm AuNPs functionalized with redox mediator molecules (NQ), coated on our lab-made screen-printed CNT-modified electrode (Figure 1). This enzyme-based electrode was based on glucose oxidation catalyzed by GOx with the help of NQ-mediated AuNPs in the matrix of egg white proteins.

We modified a screen-printable ink by adding CNTs to conductive ink due to their high conductive properties and ability to act as efficient current collectors. The addition of CNTs with a high aspect ratio (~700–6000) promoted the percolation of ink within its matrix, thereby facilitating the flow of electrons in electrochemical processes [32]. Then, the printed CNT-modified electrode was functionalized with a GOx enzyme, co-immobilized with egg white proteins and NQ-AuNPs, to enhance the enzyme stability, conductivity, and adsorption surface (Figure 1A).

GOx from *Aspergillus niger* is a flavoprotein that utilized molecular oxygen as an electron acceptor in GOx-catalyzed oxidation of β-D-glucose at its linked hydroxyl group to produce gluconolactone and hydrogen peroxide. It is recognized as an ideal enzyme for biosensor application due to its high specificity toward β-D-glucose, stability, high activity, and commercial availability [33]. Due to the advantages of GOx enzyme, biosensing has been shown to be a revolutionary approach in various fields in recent decades, ranging from environmental to biomedical applications, including diabetes control.

The first generations of biosensors that measure the concentration of the depletion of oxygen or the products of enzymatic processes were developed. This class of biosensors is oxygen-dependent, which relies on the use of natural oxygen. Glucose concentration is determined by following the consumption of oxygen or the generation of hydrogen peroxide [33,34]. However, the major obstacles of first-generation electrochemical biosensors in analyzing real samples are the interference of electroactive species (such as ascorbic acid) and the effect of oxygen. This difficulty has been tackled with a number of strategies.

One of the most useful strategies is to design an efficient mediated electrochemical biosensor, which is a second-generation biosensor. This class utilizes mediators as redox agents to act as electron carriers, thus replacing the oxygen in the reaction. For the GOx-based electrode, movement of electrons specifically occurs when the GOx enzyme catalyzes the oxidation of glucose to gluconolactone via the reduction of the FAD, the redox center of GOx, to FADH_2_ [35]. However, the 3D structure of GOx reveals that FAD (redox cofactor) is deeply buried within the protein shell. Thus, the electron transfer between the GOx active site and the electrode surface in GOx-based biosensors is limited by a thick protein layer of GOx surrounding a FAD center. This prevents the electron from communicating directly with the electrode surface. Since the electron transfer from the FAD active site of GOx to the electrode surface is challenging, the use of a mediator molecule can help to shuttle electrons, which is called mediated electron transfer (MET) [4]. Hence, we designed the mediator-based glucose biosensor in this study to enhance electron transfer, reduce interference effects, and mitigate the influence of oxygen. We utilized NQ as a redox mediator to transport electrons between the FAD center of GOx and the electrode surface. In general, quinones are a family of carbonyl compounds with two carbonyl groups in a six-member ring structure. Due to their excellent electrochemical reversibility, rapid redox kinetics, stable structure, and low molecular weight (158.16 g mol^–1^), redox-active quinones compounds have the potential to rapidly transfer electrons from the enzyme redox center to our CNT-modified working electrode surface, achieving high-rate capability and long-term cycle stability [36,37]. However, only the immobilization of NQ on the electrode by physical absorption or noncovalent interactions can lead to leaching from the electrode surface [38]. Thus, a conductive nano-matrix with CNTs and AuNPs is an important material for NQ immobilization. NQ can be strongly integrated with the bottom layer of the CNT-based electrode since NQ can interact with a CNT-modified electrode by means of π–π interactions between the aromatic groups of quinones and CNT surfaces [37]. The electrical wiring from the FAD center to CNTs with NQ as a linker is possible for enhancing the electron-transfer rate. In addition to using NQ, we additionally connected AuNPs with this mediator. The possible interaction between NQ and AuNPs could involve adsorption. The surface-to-volume ratio of a AuNP in this work was 1.2 nm^2^/nm^3^. The increase in active surface area induced by the presence of conductor nanomaterials was expected to improve electron transfer kinetics, resulting in improved glucose-sensing sensitivity and power density for BFCs.

However, a key barrier to achieving stable biosensors is the problems associated with enzyme immobilization. Thus, the stability of enzymes is important for enzyme-based glucose sensors. In conventional devices, substrates for immobilizing enzyme are often conducting polymers, semiconductors, metals, and carbon-based materials, which have some disadvantages, including high cost, potential environmental risks, and non-renewability [8].

In this work, raw egg white proteins (mainly ovalbumin) were employed as an immobilization matrix to entrap the GOx enzyme. Ovalbumin is a unique protein biopolymer, consisting of both hydrophobic domains (at both ends of the chain) and hydrophilic domains (at the middle of the chain) [39]. As shown in Supplementary Figure S1, the peptide chain of ovalbumin, with a molecular weight of about 45 kDa, consists of 386 amino acids, such as lysine residues, glycine, and proline [40], which offer a large number of functional groups such as −COOH and −NH_2_. Due to having both hydrophilic and hydrophobic regions, the protein in egg whites can readily undergo a conformational transition between a water-soluble structure and a water-insoluble structure in response to changes in temperature or chemicals [41]. Additionally, the availability of amino acids is important for the glutaraldehyde crosslinking process. The GOx enzymatic network formed by cross-linking egg white proteins serves as 3D skeletons to which GOx could be linked by glutaraldehyde, resulting in a large scaffold for biochemical reactions with immobilized enzymes (Supplementary Figure S2). It was shown that the amide bond of the NH_2_ molecule, when it came in contact with the carbonyl group of glutaraldehyde, could react to form the –N=C– bond by losing a molecule of water [42]. In this way, egg white proteins crosslinked with glutaraldehyde were formed as a 3D skeleton. The Fourier-transform infrared spectroscopy (FTIR) was further conducted to examine functional groups and the bonding information of egg white proteins with and without glutaraldehyde crosslinking (Supplementary Figure S3). Specifically, the FTIR spectra of egg white proteins without glutaraldehyde showed a peak at 1550 cm^–1^, indicating the presence of the N–H bond, which is a characteristic functional group of proteins. However, the intensity of this peak was reduced when egg white proteins were crosslinked with glutaraldehyde. The C–H stretching vibration (at around 2959 cm^–1^) and the peak of C–C bonding (at around 1004 cm^–1^) were more prominent when crosslinking with egg white proteins. This suggested the successful crosslinking reaction between egg white proteins and glutaraldehyde. Both the interaction between binding sites of egg white proteins and enzyme and a porous structure formed during the crosslinking process of protein can serve as a skeleton for the immobilization of enzyme, preventing the enzyme from leaking off the electrode surface and ensuring the sensor’s stability [9].

Importantly, biocompatible bioelectronic devices are of particularly great interest regarding biomedical applications. In comparison with other commercial proteins, egg white proteins are less expensive. For example, egg white proteins cost only 0.003 USD per 1 g, whereas BSA cost much more (25 USD per 1 g). Since egg white is safe, biocompatible, and cheap, these characteristics make it a preferable choice as a material for sensing applications, such as a stabilizer of nanoclusters [43], a hydrogel crosslinker [44], and a coating layer of nanoparticles [45], and also for modern applications such as wearable devices and soft bioelectronics that have non-inflammatory contact with human tissue. Therefore, with this strategy of enzyme immobilization, our design of an enzyme-based electrode has a stable biosensor structure.

For the kinetic of the redox mediator-modified nanoparticles–enzymatic proteins interaction, an electron mediator should be capable of efficiently facilitating electron transfer at a specific potential and must be able to transfer electrons rapidly. The study of electrochemical enzyme kinetics of redox-mediated GOx reactions is important since it can help determine the enzyme’s affinity to the substrate (glucose) and its maximum catalytic efficiency. The Michaelis–Menten constant (*K_m_*) is an important enzyme-substrate kinetic indicator of the corresponding enzyme [46]. In the heterogeneous system, we investigated the interaction between GOx and glucose (substrate) using an amperometric technique to observe the number of generated electrons per time unit (Figure 1B). In electrochemical kinetics, the current output can be correlated to the reaction rate with successive additions of the substrate. The dependence of the current responses on substrate concentration showed the characteristics of the Michaelis–Menten kinetic mechanism (inset of Figure 1B). For enzymes that conformed to the Michaelis–Menten mechanism, the early phase of increasing substrate concentrations results in a rapid increase in current, followed by a gradual increase as the enzyme approached its maximum activity. Due to enzyme saturation, the maximum current achieved at high substrate concentrations is fully in enzyme-substrate complex form. Thus, the curve depicts the kinetic parameters defining the curve’s high and low substrate concentration boundaries.

In this article, we also demonstrated the working operation for energy-harvesting and self-powered sensing modules by coupling these egg white proteins/GOx/NQ-AuNPs/CNT-modified bioelectrodes with a printed Pt-based cathode (Figure 1C). The reaction occurring on the bioanode was based on glucose oxidation catalyzed by GOx, resulting in electrons being released. These electrons went through the power load of the self-powered biosensing unit toward the Pt-based cathode; eventually, natural oxygen reached the cathode to gain the electrons, completing the power circuit. Our BFC (using redox-mediated AuNPs with GOx immobilized by egg white proteins on the anode and the Pt/CNT-based catalysts on the cathode) could convert chemical energy in glucose molecules into electrical energy in the form of electricity (Figure 1C, right-top). We also observed that this electrical energy produced by redox reactions could be proportional to the concentration of glucose. Consequently, the screen-printable device can also function as a self-powered biosensor capable of measuring glucose concentration without the need of an external energy supply to force the glucose oxidation (Figure 1C, right-bottom).

### 2.2. The Effect of AuNPs on Glucose Oxidation Kinetics

**Figure 2:**
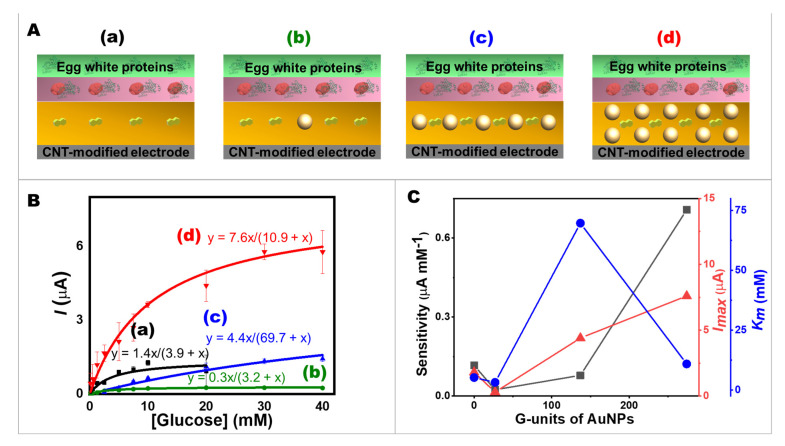
Effects of AuNPs on glucose oxidation kinetics. (**A**) The components of screen-printed bioelectrodes with (**a**–**d**) 0, 27, 137, and 274 G-units of AuNPs and 750 nmol NQ. (**B**) The calibration plots. (**C**) Comparison of the bioelectrode performances in terms of sensitivity, *I_max_*, and *K_m_* values.

The effect of AuNPs on glucose oxidation kinetics at the four different modifications of the CNT-based screen-printed bioelectrodes using amperometry was studied in terms of sensitivity, *I_max,_* and *K_m_*. Figure 2 illustrates the four different approaches of electrode modification based on the different number of AuNPs, including 0, 27, 137, and 274 G-units in a matrix of NQ (750 nmol), and the enzyme layer (25 units of GOx) with the egg protein layer were controlled (Figure 2A). The amperometric responses of the different modified screen-printed bioelectrodes were recorded at different glucose concentrations in a range of 0.0 to 40 mM by holding a potential of 0.40 V vs. Ag/AgCl (Supplementary Figure S4). Figure 2B shows the dependence of current response on glucose substrate concentrate ion, which agreed with the Michaelis–Menten equation (Equation (1)). This curve shows that currents increased rapidly after glucose addition, and slowly before reaching their limit. The maximal current at high glucose concentrations was due to saturation. Adding more glucose would no longer increase the current. The calibration plots in Figure 2B (a–d) of the CNT screen-printed electrodes with AuNPs incorporated in a NQ matrix revealed that the current response of the 274 G-unit of AuNPs-modified bioelectrode (trace d), which had twice and 10-times AuNPs higher than Electrode c and Electrode b showed the highest current signals, followed by 137 and 27 G-unit of AuNPs (c and b), respectively.

(1) $$I=\frac{I_{max}\left[C\right]}{K_{m}+\;\left[C\right]}\;$$

where I is the steady-state current after the addition of glucose, $I_{max}$ is the maximum current obtained from saturated glucose concentrations, *C* is the glucose concentration, and *K_m_* is the Michaelis–Menten constant.

Additionally, a supporting equation presented the double reciprocal relationship following the Lineweaver–Burk equation (Equation (S1)) which was algebraically transformed from the Michaelis–Menten equation. Moreover, the Hanes–Woolf equation (Equation (S2)), and the Eadie–Hofstee (Equation (S3)) were also used to calculate *K_m_* and *I_max_* values (see details in Supporting Information). The *K_m_* and *I_max_* from straight lines of the double reciprocal plot, the Hanes–Woolf plot, and the Eadie–Hofstee plot were also demonstrated (Supplementary Figure S5).

The parameters of enzymatic kinetics are shown in Figure 2C. When using NQ-AuNPs, the *K_m_* value could reach ~3 mM, indicating good apparent affinity of GOx immobilized on the bioelectrode to the glucose target. Regarding the ratio of AuNPs and NQ molecules, the results indicated that highly conductive AuNPs offered a high specific surface area for GOx. Hence, the number of AuNPs had a clear impact on the analytical performances of the glucose. The greater the number of AuNPs, the higher *I_max_* and higher sensitivity were obtained. Regarding the effect of AuNPs on the *I_max_*/*K_m_* ratio, it appears that the use of AuNPs on Electrode d leads to a significantly higher *I_max_*/*K_m_* ratio compared to Electrode a. The *I_max_*/*K_m_* ratio was an indicator of the catalytic efficiency of an enzyme. The 2.1 to 3.0 times increase in the *I_max_*/*K_m_* ratio of Electrode d compared to Electrode a, as calculated from various kinetics models (Michaelis–Menten, Lineweaver–Burk, Hanes–Woolf, and Eadie–Hofstee plots), further confirms the advantages of using AuNPs in electrochemical sensing. Interestingly, the *I_max_* response of the egg white proteins/GOx/NQ-based bioelectrodes without AuNPs (Electrode a in Figure 2) was higher than Electrode b (27 G-units of AuNPs). However, these current signals of Electrode a saturated at a lower concentration (at 10 mM glucose). The sensitivity of Electrode a was higher than Electrodes b and c. However, a maximum current of Electrode c was higher than electrode a. This might be because the small amount of AuNPs dispersed in 0.1 mg mL^−1^ sodium citrate could attribute the AuNPs-NQ surface to be a negative charge, while the GOx had a negative charge at pH 7.0 [47]. This could lead to repelling each other, resulting in the observation that Electrode b displayed a lower maximum current response than Electrode a.

### 2.3. The Effect of Layout Arrangements on Glucose Oxidation Kinetics

**Figure 3:**
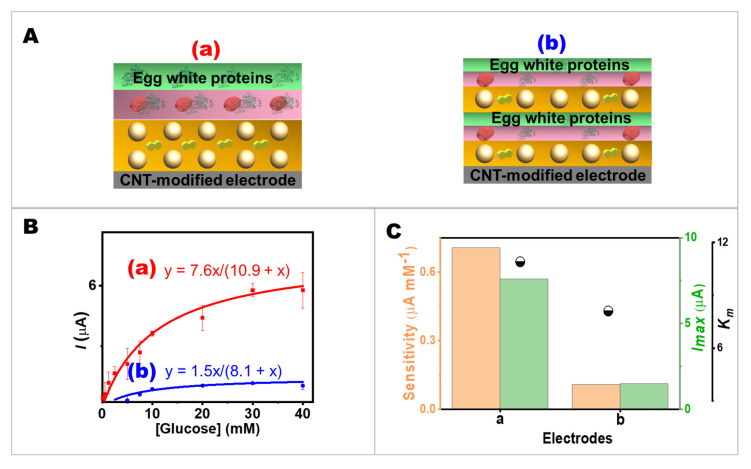
Effects of layout arrangements on glucose oxidation kinetics. (**A**) The components of a screen-printed bioelectrode in (**a**,**b**) two different layout arrangements with the same total amount of 10 µL of 10% *v*/*v* egg white proteins, 10 µL of 10 mg mL^−1^ GOx (in 10% *v*/*v* egg white proteins), and 274 G-units of AuNPs with 750 nmol NQ. (**B**) The calibration plot of the current response. (**C**) Comparison of the bioelectrode performances in terms of sensitivity, *I_max_*, and *K_m_* values.

The effect of layout arrangements on glucose oxidation kinetics at the two different modifications of the layers was studied. Figure 3A illustrates the comparison of electrode modification based on the different layer arrangement while maintaining a constant total volume of 10 µL of 10% *v*/*v* egg white proteins, 10 µL of 10 mg mL^−1^ GOx (in 10% *v*/*v* egg white proteins), and 274 G-units of AuNPs with 750 nmol of NQ). The amperometric responses of the different modified screen-printed electrodes (Supplementary Figure S6) were performed at different glucose concentrations in the range of up to 40 mM. The calibration plots of two different layout arrangements were demonstrated in Figure 3B. Additionally, the double reciprocal plot of the calibration curve was obtained from screen-printed bioelectrodes (Supplementary Figure S7). It is clearly seen from the curve that Electrode b showed a lower *I_max_* than Electrode a. Considering the sensitivity, *I_max_*, and *K_m_* values, the effectiveness of the layout arrangement could be evaluated (Figure 3C). Because Electrode b had lower sensitivity and *I_max_* than Electrode a, it confirmed that the layout arrangement of the bioelectrode is important. As discussed earlier, the GOx layer needed to be arranged close to the redox-mediated AuNPs to assist the electron transfer from the enzyme redox center to the electrode surface. This study confirmed that our design of the bioelectrode layering is suitable for further development of advanced biosensing.

### 2.4. The Effect of the Thickness of Egg White Proteins on Glucose Oxidation Kinetics

**Figure 4:**
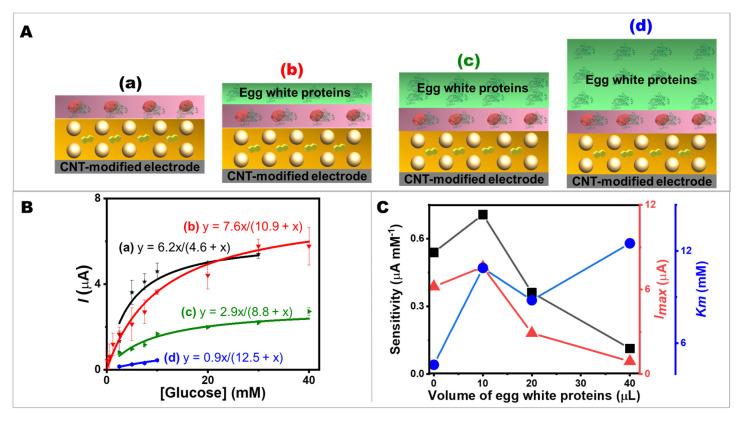
Effects of the thickness of egg white proteins on glucose oxidation kinetics. (**A**) The components of screen-printed bioelectrodes with (**a**–**d**) 0, 10, 20, and 40 µL of egg white solution on the topmost layer. (**B**) The corresponding calibration plots. (**C**) Comparison of the bioelectrode performances in terms of sensitivity, *I_max_*, and *K_m_* value.

It is important to secure the enzyme immobilized on the electrode surface with the highest possible specific activity. Immediately adjacent to the NQ-AuNPs-mediated CNT-modified electrode was the GOx layer, which was formed by the entrapment of GOx within the protein matrix by covalent glutaraldehyde cross-linking with egg white proteins. The protein as an immobilization matrix could prevent the leakage of GOx into the analyte solution and act as a diffusional barrier for the substrate. A carefully determined protein layer thickness is also advantageous to tune biosensor performances. Therefore, the effect of the thickness of egg white proteins on glucose oxidation kinetics was investigated. Figure 4A shows the effect of the variation of egg white proteins solution (0, 10, 20, and 40 µL) on the performance of glucose sensing (amperometric current response to glucose addition). As a reference, the bioelectrode without the egg white proteins solution on the top layer was fabricated (Figure 4A(a)). The calibration plots are shown in Figure 4B. The increase in thickness of the egg white proteins layer resulted in a lower maximum current and a lower sensitivity, as shown in Figure 4C. This may have been due to the porosity of the outermost surface (the layer of egg white proteins); the thicker protein matrix layer increased the diffusion length, resulting in slower glucose molecule diffusion. However, this finding is also advantageous. Most enzymes conform to the Michaelis–Menten kinetic, in which the reaction is largely non-linear with substrate addition. The thicker protein layer could broaden the linear range of the substrate’s detection. In addition to extending the linear range, the thicker layer can also protect the catalytic enzyme. A porous surface of egg white proteins protects the GOx layer from leaching out while allowing glucose to diffuse to the enzymatic layer. Adjusting the thickness of the protein layer allows a flexible screen-printed CNT-based bioelectrode to detect glucose over an adjustable range, as the response is controlled by diffusion through the egg white proteins’ matrix and not solely enzymatic kinetics. This design allows biosensors to detect glucose efficiently since glucose could be easily diffused into the outermost layer. The good porosity of the egg-protein matrix could be evaluated by considering the sensitivity, *I_max_*, and *K_m_* values. Electrode a (without the topmost layer of egg white proteins) and Electrode b (with the topmost layer of egg white proteins) had similar *I_max_* values, but Electrode a had a lower *K_m_* value. This may have been because the presence of egg white proteins on the surface decreased the apparent affinity of GOx. However, both Electrode a and b showed higher current values than Electrode c and d, respectively. Since the small *K_m_* value confirmed the high affinity of the GOx immobilized on the bioelectrode, it was apparent from the Electrode a and b (i.e., 0 and 10 µL of egg white solution, respectively) that having a protein layer with only a small amount had no major effect on the accessibility of glucose to GOx. It could still maintain the small value of *K_m_* and a good sensitivity while offering a bio-friendly environment for GOx. The configuration of the protein layer on the NQ-AuNPs-mediated CNT-modified electrodes, which is a simple way to modulate analytical performances (sensitivity and linear range), enabled the bioelectrodes to be used to measure glucose concentration in a desirable range of analytical substances.

### 2.5. The Effect of Egg White Proteins on Operational Stability

**Figure 5:**
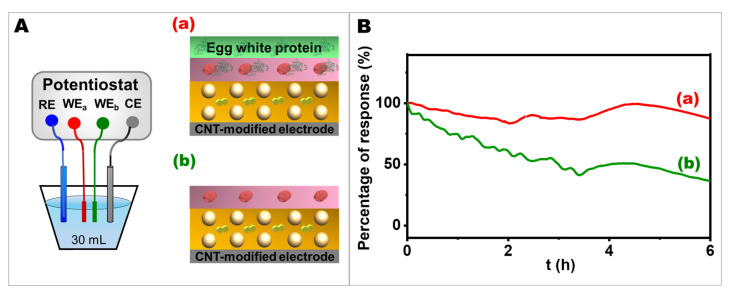
(**A**) The electrochemical setup and electrode configurations. (**B**) Response curves of the different bioelectrodes to 10 mM glucose when operating continuously in 0.1 PBS for 6 h by applying a constant potential of 0.4 V (vs. Ag/AgCl). (**a**) The bioelectrode contains egg white proteins. (**b**) The control had no egg white proteins.

The operational stability of the electrode was a particular concern in terms of its future applications. In Figure 5, the operational stability of the device was evaluated in a batch system for 6 h in 0.1 M phosphate buffer solution (PBS), pH 7.0, containing 10 mM glucose to demonstrate the feasibility of the bioelectrode in a continuous operation mode. The electrodes with and without egg white proteins in their layer configuration (Figure 5A(a) and Figure 5A(b), respectively) were compared to determine the influence of egg white proteins matrix on electrode stability. Under a constant potential of 0.40 V vs. Ag/AgCl, it is clearly seen from Figure 5B that the electrode with the egg white proteins matrix at the outermost layer possessed a stable current response throughout 6 h period. For the first three hours, the current response decreased to about 87% and 47% for the electrode with and without egg white proteins, respectively, while for the last three hours, the current response showed overall stability because at the 6th h, it went up and down to almost the same value as at the 3rd h. Overall, the electrode with egg white proteins showed only about a 12% decrease from the initial stage, while the electrode without the egg white proteins matrix faced a sharp decline in the current response, ending with less than half of its initial ability after 6 h of measurement. The bioelectrode consisting of the NQ-AuNPs nanocomposite film and porous structure formed during the crosslinking process of egg white proteins with GOx could firmly hold enzyme molecules and facilitate electron flow between the redox center of the enzyme and the electrode surface, resulting in a more stable sensing performance than the electrode structure without a protein matrix layer. Moreover, as shown in Supplementary Figure S2, there were a large number of amino acids in egg white proteins; thus, a large scaffold for biochemical reactions can be formed by the crosslinking between egg white proteins’ amino acids and GOx, and also between GOx and glutaraldehyde. This 3D skeleton could prevent the enzyme from leaking off the electrode surface, resulting in the stability of the electrode.

Enzyme immobilization with other protein matrices was also reported to protect the enzyme’s activity while enhancing the electrode’s stability. Similarly, the use of silk fibroin nanofibrils, a natural protein in arthropods, was shown to improve operational stability since its structure is based on nanoporous enzymatic membranes formed by the cross-linking of silk fibroin nanofibrils with GOx [9]. To construct a cross-linked matrix to protect enzymes, BSA was also reported to be used as co-immobilization matrix with glutaraldehyde for the hydrogen peroxide sensor [5]. Unlike other immobilization materials, our bioelectrodes used low-cost, highly biocompatible food-based biomolecules to hold enzymes on nanoparticle-based bioelectrodes, which provide an advantage in further applications, such as wearable and flexible devices that should be compatible with human skin. Our result confirmed that the electrode with egg white proteins could function as an immobilization matrix to entrap enzymes within the electrode surface without leaking off into the solution, and the electrode was still functional after many hours of continuous operation.

### 2.6. Studies of a BFC and a Self-Powered Sensor Using a Screen-Printed Egg White Proteins/GOx-Egg White Proteins/NQ-AuNPs-Based Bioelectrode and a Pt-Based Cathode

With the concept of flexible bioelectronics, many useful and versatile applications are possible, including wearables [48]. However, the developed bioelectrodes should be compatible with human skin for safety reasons. Enzymatic BFCs, which transform the biological energy available in human biofluids into electricity, are one of the most potent energy generation alternatives, as a sustainable energy source for flexible bioelectronics. Because of their benefits for operation with enzymes that are active at room temperature and under mild physiological circumstances, enzymatic BFC is good for contact with human skin, enabling on-body applications [49]. BFCs, in addition to being energy-conversion devices, can be utilized as self-powered electrochemical biosensors to detect analytes without the need for external power. The oxidation reaction occurs at the bioanode when glucose interacts with the active site of GOx, whereas the reduction reaction occurs at the cathode. The bioanode and the cathode have different electrical potentials, resulting in the flow of electrons. Therefore, we aimed to evaluate our BFC to confirm its functions as an energy harvester and as a self-powered sensing device.

**Figure 6:**
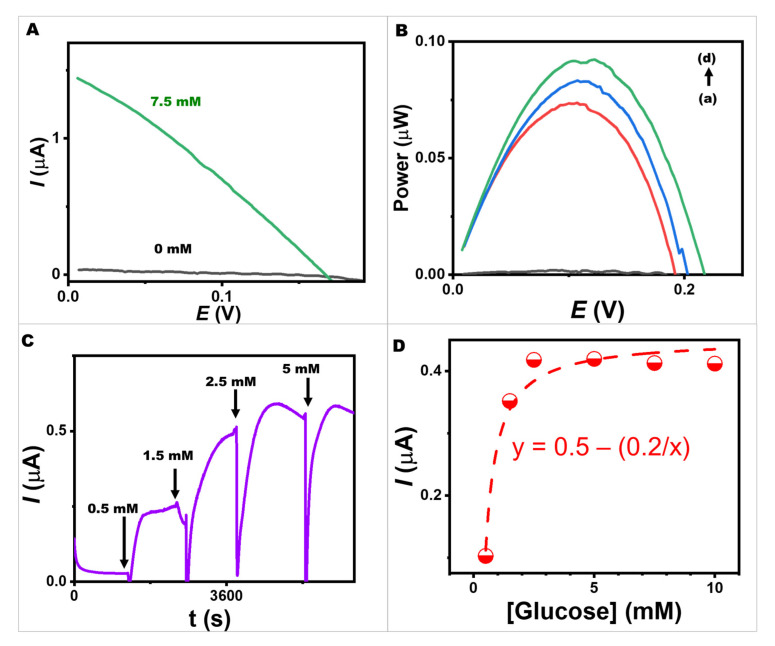
A BFC and a self-powered biosensor using a screen-printed egg white proteins/GOx-egg white proteins/NQ-AuNPs-based bioelectrode coupled with a Pt-based electrode. (**A**) Polarization curves of a screen-printed egg white proteins/GOx-egg white proteins/NQ-AuNPs-based bioelectrode coupled with a Pt-based electrode at blank (black line) and 7.5 mM (green line) of glucose concentrations in 0.1 M PBS, pH 7.0. (**B**) Power density versus potential plots of a screen-printed glucose BFC at different glucose concentrations ((**a**–**d**): 0, 2.5, 5.0, and 7.5 mM) in 0.1 M PBS, pH 7.0. (**C**) Current response of the BFC with no applied potential upon increasing the glucose concentrations in 0.1 M PBS, pH 7.0. (**D**) The corresponding calibration plot of the current response of the BFC.

The energy conversion performance of the fully functionalized BFCs using the bioanode (10 µL of 10% *v*/*v* egg white proteins, 10 µL of 10 mg mL^−1^ GOx in 10% *v*/*v* egg white proteins, and 274 G-units of AuNPs with 750 nmol of NQ) and a printed Pt-based cathode is shown in Figure 6A,B. The BFC’s performance was tested in different concentrations of glucose under pH 7.0 and ambient conditions. Various potential differences were applied between the bioanode and cathode to evaluate the BFC performance at varying glucose concentrations. A lab-made screen-printed egg white proteins/GOx-egg white proteins/NQ-AuNPs/CNT-modified bioelectrode coupled with the Pt-based cathode provided an open circuit voltage of 220 mV, a maximum output density of 0.9 μW, and a maximum current density of 1.4 μA. The power output was linearly proportional to the analyte concentration in the low glucose concentration range and reached a plateau at high concentrations. Self-powered glucose biosensors can benefit from this characteristic for further development.

The screen-printed glucose BFC was investigated as a self-powered glucose biosensor. The short-circuit method was employed to obtain self-powered detection without the requirement of any externally applied potential. The current response generated by the BFC itself was observed with successive additions of glucose ranging from 0 to 5 mM (Figure 6C,D). The saturation attained at 5 mM could be because the cathode was a limiting factor for our BFC. In the BFC mode, the bioanode uses GOx to catalyze the oxidation of glucose to generate electrons. These electrons are then transferred to the cathode, where the oxygen reduction reaction (ORR) occurs to consume the electrons and protons to form water [49,50], resulting in the generation of the electric current. In this study, the glucose oxidase-based bioelectrode acted as the bioanode, while the Pt-based electrode acted as the cathode. On the cathode surface, the rate of ORR could be limited by several factors, including the concentration of oxygen and the availability of active sites on the cathode surface. At high glucose concentrations of 5 mM, the bioanode produced a large number of electrons, which needed to be consumed by the cathode. In our BFC system, the rate of ORR on the cathode surface may not be able to keep up with the rate of electron and proton production on the bioanode; thus, the overall current output was limited. However, without external power or potentiostat, glucose could be sensed using our self-powered BFC consisting of the egg white proteins/GOx-egg white proteins/NQ-AuNPs-based bioelectrode. Moreover, the device was expected to run in a stand-alone mode because the generated power was proportional to the analyte concentration.

## 3. Materials and Methods

### 3.1. Chemicals and Materials

Multiwalled CNTs (95% purity, diameter = 5–15 nm, length = 10–30 μm) were from Luoyang advanced material Co., Ltd., Shanghai, China. D-(+)-glucose anhydrous was from Fluka, Neu-Ulm, Germany. Gox (from *Aspergillus niger*, type VII, >100,000 units g^–1^), NQ, glutaraldehyde solution (grade II, 25% in H_2_O), platinum black, and Nafion-117 solution were from Sigma-Aldrich, (Saint Louis, MO, USA). Potassium phosphate dibasic (K_2_HPO_4_) and potassium phosphate monobasic (KH_2_PO_4_) were from BDH Laboratory Supplies Poole, UK. Sodium carbonate (Na_2_CO_3_) was from Ajax Finechem, (New South Wales, Australia). Tetrahydrofuran (THF) was from Honeywell, International Inc., (Charlotte, NC, USA). Toluene was from Guangdong Guanghua Chemical Factory Co., Ltd., Guangdong, China. AuNPs (5 nm in 0.1 mg mL^−1^ sodium citrate with stabilizer, 5.47 × 10^13^ particles mL^−1^) were from Thermo Fisher Scientific Waltham, MA, USA. Ethanol was from RCl Labscan Ltd., (Bangkok, Thailand). Acetone was from VWR International Ltd., (Poole, UK. Chicken eggs (large size, weight 63–73 g) were brought from Charoen Pokphand Foods PCL, (Bangkok, Thailand). All chemical solutions were prepared using ultrapure deionized water (18.2 MΩ cm) from a Milli Q Merck system, (Darmstadt, Germany). 0.1 M PBS was produced with a pH value of 7.0 and used as the supporting electrolyte.

### 3.2. Instruments and Electrochemical Measurement

The electrochemical performance of a screen-printed bioelectrode was evaluated in 0.1 M PBS, pH 7.0. A potentiostat/galvanostat (Autolab Type III FRA 2, Metrohm) and µStat-I 400 (Dropsens, Metrohm) were used to examine electrochemical performances, including linear sweep voltammetry and amperometry. Working electrodes (such as screen-printed egg white proteins/GOx/NQ-AuNPs/CNT-modified electrodes) were evaluated with Pt counters and Ag/AgCl (3.0 M KCl) reference electrodes during the three-electrode-system analysis. The amperometric response was measured, after 30 s immersion in the test solution, by stepping the potential to 0.40 V (vs. Ag/AgCl) for 30 s. Calibration plots were obtained by increasing the glucose concentration in 0.1 M PBS, pH 7.0. We set a two-electrode system to determine the BFC performances as an energy harvester and as a self-powered sensing device. Open circuit voltage (OCV) was assessed prior to recording a polarization plot. We recorded the BFC signal’s polarization curve between OCV and 0 V. The scan rate for recording the curve was 1 mV s^–1^. The autonomous biosensing method was recorded using a digital multimeter (Keysight model 34465A) coupled with Keysight BenchVue Software. The short-circuit method was employed to obtain self-powered detection. Infrared spectra (IR) were measured by an FTS165 FTIR spectrometer.

### 3.3. Preparation of CNT-Modified Carbon Ink

To prepare a CNT-modified ink, 30 mg of CNTs were dispersed in 490 µL of toluene using a homogenizer probe (model AR-0975) level 1 for 3 min. Next, 7 g of the carbon conductive ink (Guangzhou Print Area Technology Co. Ltd., Guangzhou, China), was mixed with the mixture using a mixing machine (Shashin Kagaku, Kakuhunter, SK-300SII, Japan) for 30 min at 2000 rpm. The resulting CNT-modified carbon ink was printed on PET as a working electrode. In this study, the area of the working electrode was 0.3 cm × 0.5 cm.

### 3.4. Egg White Proteins/GOx/NQ-AuNPs-Based Immobilization

The printed CNT-modified carbon electrode was cleaned with 1 M Na_2_CO_3_ at a potential of 1.5 V for 60 s. The CNT-modified carbon electrode was functionalized by dropping 10 µL of NQ-AuNPs (prepared by mixing 5 µL of AuNPs solution with 5 µL of 150 mM NQ dissolved in ethanol/acetone mixture (a volume ratio of 4:1)). In other words, this resulting electrode was coated with 274 G-units of AuNPs and 750 nmol of NQ. The dropped layer was left to dry at room temperature. Next, 10 µL of 10 mg mL^−1^ GOx (dissolved in 10% *v*/*v* of egg white solution in 0.05 M PBS, pH 7.0) was dropped on an electrode surface. After drying at room temperature, 10 µL of 10 % *v*/*v* of egg white in 0.05 M PBS, pH 7.0 was dropped above NQ-AuNPs layer. Then, 10 µL of 0.75 wt% glutaraldehyde was dropped on the bioelectrode and left to dry at room temperature. Additionally, different electrode configurations were prepared to investigate different enzyme kinetics of glucose oxidation. Effects of AuNPs dispersion were studied by varying numbers of particles at 0, 27, 137, and 274 G-units of AuNPs with a constant amount of NQ (750 nmol). The different molarities of AuNPs in the AuNPs/NQ mixture were controlled to be 0, 4.54, 22.7, and 45.4 nM, while the molarity of NQ in the AuNPs/NQ mixture was controlled to be 75 mM to achieve different numbers of AuNPs per one electrode (0, 27, 137, and 274 G-units, respectively). The effect of layer was studied by varying the arrangement of the component of egg white proteins/GOx/NQ-AuNPs on screen-printed bioelectrodes which maintained a total volume of 10 µL of egg white proteins, 10 µL of 10 mg mL^−1^ GOx in 10% *v*/*v* egg white proteins, and 274 G-units of AuNPs with 750 nmol NQ. The effect of the thickness of egg white proteins was studied by varying volumes of egg white proteins at 10, 20, and 40 µL of 10% *v*/*v* egg white solution.

### 3.5. GOx/NQ-AuNPs-Based Immobilization without Egg White Proteins

To set a control experiment, the modified bioelectrode without egg white proteins coated on the outermost layer was prepared. The control electrode was coated with 10 µL of NQ-AuNPs (prepared by mixing 5 µL of AuNPs solution with 5 µL of 150 mM NQ). Next, 10 µL of 10 mg mL^−1^ GOx (dissolved in 0.05 M PBS, pH 7.0) was dropped on NQ-AuNPs/CNT-modified bioelectrode. Then, 10 µL of 0.75 wt% glutaraldehyde was dropped on the bioelectrode and left to dry at room temperature.

### 3.6. Preparation of a Pt-Based Cathode

The cathode was covered with a layer of carbon ink modified with CNTs. The cathode was fabricated by drop casting solution of 5 μL of 5 mg mL^−1^ CNTs dispersed in 10 mg mL^−1^ platinum black in ethanol solution. Then, 3 µL of 0.5% Nafion ^®^ solution was dropped and dried overnight at room temperature.

## 4. Conclusions

We have presented a new approach to fabricating a glucose amperometric biosensor and a bioanode for a BFC and self-powered biosensor using redox-mediated AuNPs, GOx, and egg white proteins. This highly biocompatible and cost-effective strategy enhances the long-term stability and self-powering capabilities of the biosensor while maximizing electron flow from the redox center of GOx. The results of our study provide insight into the relationship between the immobilization of enzymes, nanoparticles, and food-based proteins and the subsequent bioelectrochemical behavior. Due to their versatility, egg white proteins are considered useful for in vitro themes as biomaterials because they are inexpensive, commercially available, and biocompatible. Vividly, AuNPs have a great impact on electrochemical performances including high sensitivity and *I_max_* because of excellent glucose oxidation currents and high surface-to-volume ratio properties. Moreover, adjusting the thickness of the egg white proteins layer may allow a wider linear range due to the diffusion limiting. In addition, through the studies of bioelectrodes, we demonstrated the continuous monitoring system in the batch cell system for 6 h utilizing the bioelectrodes with egg white proteins. We ascertained that the egg white proteins can support the stability of the bioelectrodes compared to non-egg white proteins bioelectrodes. These findings have important implications for the development of advanced biosensing and bioenergy conversion systems, as we developed the self-powered biosensor and the outcome stated that the generated power was proportional to the analyte concentrations. Herein, this flexible platform could be a promising tool as a portable and stand-alone device eliminating the need for a potentiostat. Future work should focus on studying a surface charge investigation for an in-depth understanding of AuNPs charge transferring and enhancing the performance of the cathode by engineering catalytic materials to improve ORR electrocatalysis.

## Data Availability

Data are contained within the article and the Supplementary Material.

## Supplementary Materials

The following supporting information can be downloaded at: https://www.mdpi.com/article/10.3390/ijms24054657/s1.
