# Supplementary material for: Redox-Mediated Gold Nanoparticles with Glucose Oxidase and Egg White Proteins for Printed Biosensors and Biofuel Cells
Source: Int J Mol Sci. 2023 Feb 28;24(5):4657. doi: 10.3390/ijms24054657 (PMC10002497; doi:10.3390/ijms24054657)
Supplement: Supplementary file 1 [file ijms-24-04657-s001.zip › ijms-2222968-supplementary.pdf]

Supporting Information

Redox Mediated Gold Nanoparticles with Glucose Oxidase and Egg White Proteins for Printed Biosensors and Biofuel Cells

Natcha Rasitanon[1,2], Kornautchaya Veenuttranon[1], Hnin Thandar Lwin[3], Kanyawee Kaewpradub[1,2], Tonghathai Phairatana[3,4] and Itthipon Jeerapan[1,2,5,*]

1 Center of Excellence for Trace Analysis and Biosensor, Prince of Songkla University, Hat Yai, Songkhla 90110, Thailand
2 Division of Physical Science, Faculty of Science, Prince of Songkla University, Hat Yai, Songkhla 90110, Thailand
3 Department of Biomedical Sciences and Biomedical Engineering, Faculty of Medicine, Prince of Songkla University, Hat Yai, Songkhla, 90110, Thailand
4 Institute of Biomedical Engineering, Faculty of Medicine, Prince of Songkla University, Hat Yai, Songkhla, 90110, Thailand
5 Center of Excellence for Innovation in Chemistry, Faculty of Science, Prince of Songkla University, Hat Yai, Songkhla 90110, Thailand

* Correspondence: itthipon.j@psu.ac.th
ORCID: https://orcid.org/0000-0001-8016-6411

Supporting Equations

Additional equations used for K_m and I_{max} calculation:

The Lineweaver-Burk equation:

$$\frac{1}{I} = \frac{K_m}{I_{max}}\frac{1}{[C]} + \frac{1}{I_{max}} \quad \text{(Equation S1)}$$

The Hanes-Woolf equation:

$$\frac{[C]}{I} = \frac{1}{I_{max}}[C] + \frac{K_m}{I_{max}} \quad \text{(Equation S2)}$$

The Eadie-Hofstee equation:

$$I = -K_m \frac{I}{[C]} + I_{max} \quad \text{(Equation S3)}$$

Where I is the steady-state current after the addition of glucose, I_{max} is the maximum current obtained from saturated glucose concentrations, C is the glucose concentration, and K_m is the Michaelis-Menten constant.

Supporting Figures

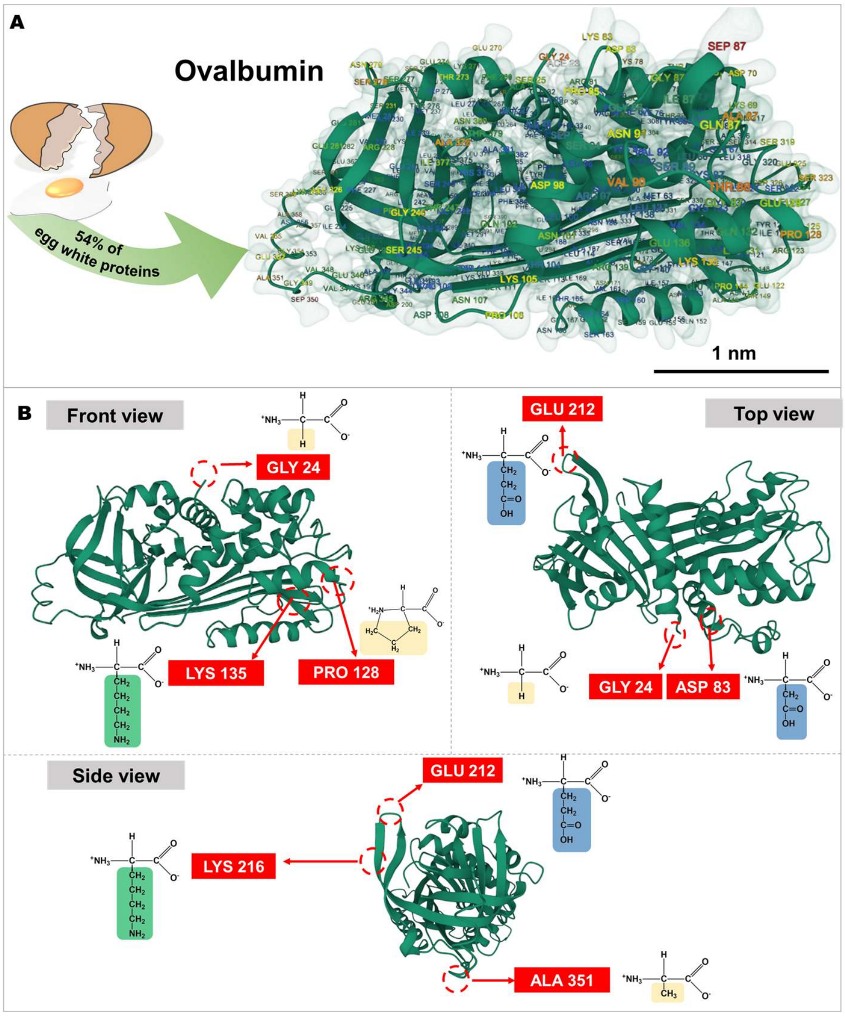

Figure S1. Structure of ovalbumin (PDB: 1OVA). (A) Overview of the ovalbumin structure and amino acids. Ovalbumin is a main component in egg white proteins [1]. (B) Location of some important amino acids, along with molecular structures, in the ovalbumin structure.

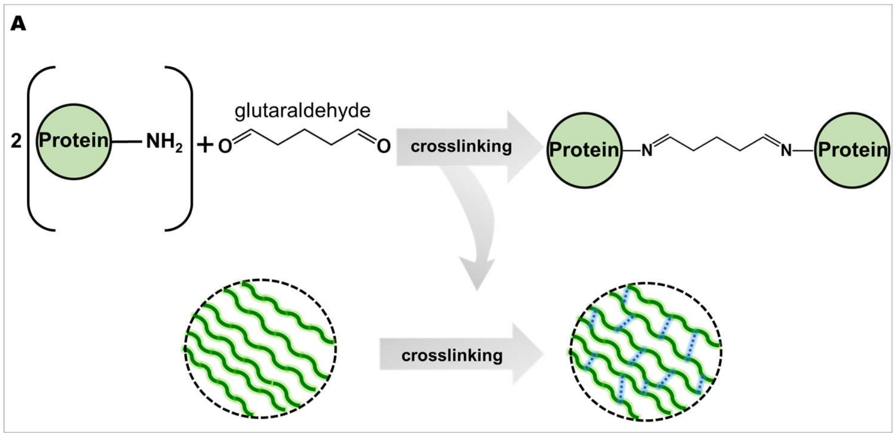

Figure S2. Cross-link reaction between proteins, such as proteins from GOx and egg white proteins (including ovalbumin), and glutaraldehyde.

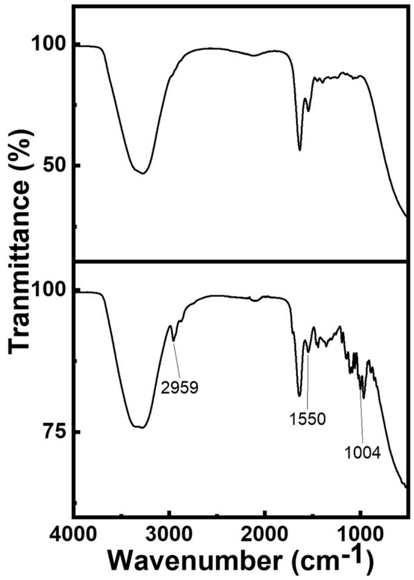

Figure S3. FTIR spectra of egg white proteins (a) before and (b) after crosslinking with glutaraldehyde.

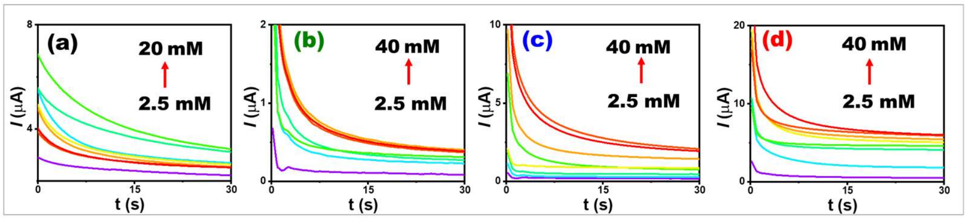

Figure S4. Amperograms of screen-printed bioelectrodes using different numbers of AuNPs with an applied potential of 0.40 V vs. Ag/AgCl upon increasing the glucose concentrations. These electrodes (a–d) refer to Figure 2A. These screen-printed bioelectrodes (a–d) use 0, 27, 137, and 274 G-units of AuNPs with 750 nmol NQ.

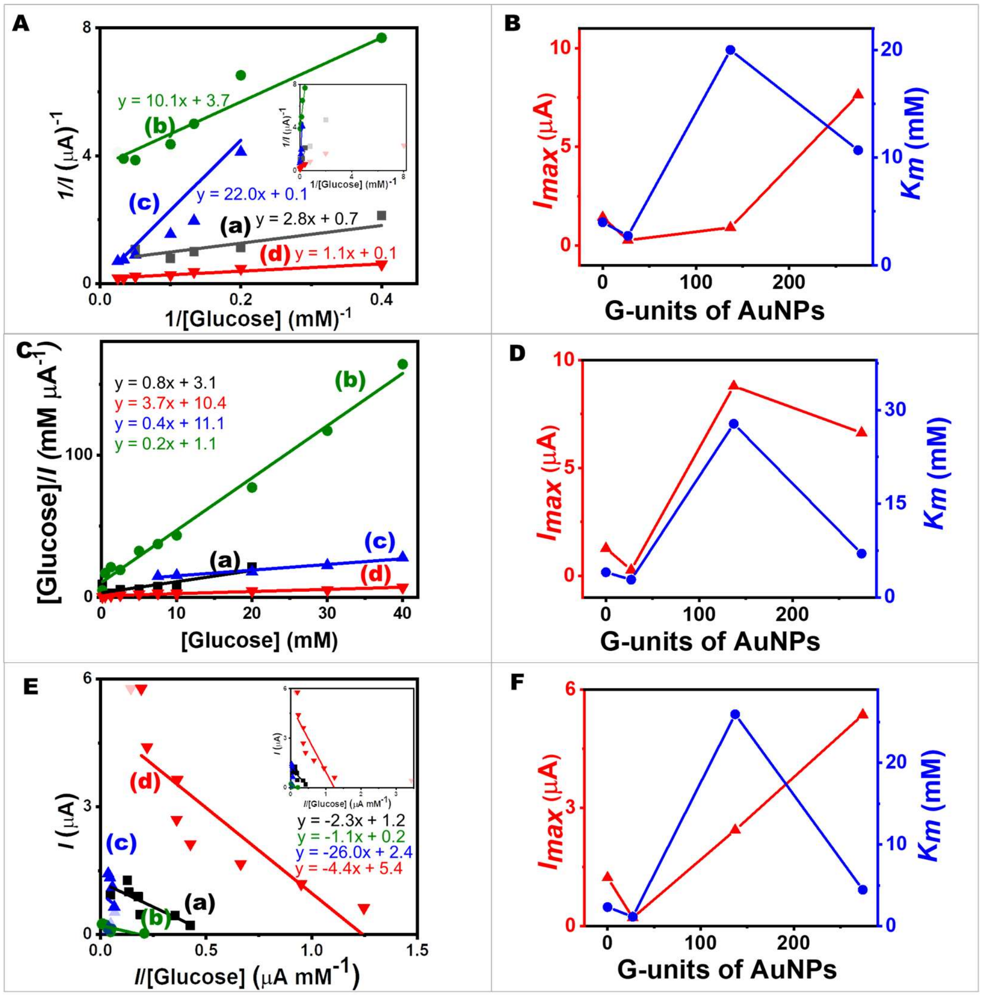

Figure S5. The plots used for estimating K_m and I_{max} referring to Figure S4. These screen-printed bioelectrodes (a–d) use 0, 27, 137, and 274 G-units of AuNPs with 750 nmol NQ. (A) The Lineweaver–Burk plot. (B) I_{max}, and K_m values estimated from the Lineweaver–Burk plot. (C) The Hanes-Woolf plot. (D) I_{max}, and K_m values estimated from the Hanes-Woolf plot. (E) The Eadie-Hofstee plot. (F) I_{max}, and K_m values estimated from the Eadie-Hofstee plot. Data points in light colors are excluded during fitting.

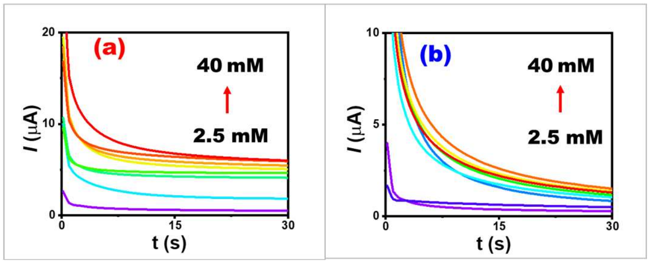

Figure S6. Amperogram responses of screen-printed bioelectrodes of different layer arrangements with an applied potential of 0.4 V vs. Ag/AgCl upon increasing the glucose concentrations. These electrodes (a–b) refer to Figure 3A. These screen-printed bioelectrodes (a–b) two different layout arrangement with the same total volume of 10 μL of 10%v/v egg white proteins, 10 μL of 10 mg mL^{-1} GOx (in 10%v/v egg white proteins), and 274 G-units of AuNPs with 750 nmol NQ.

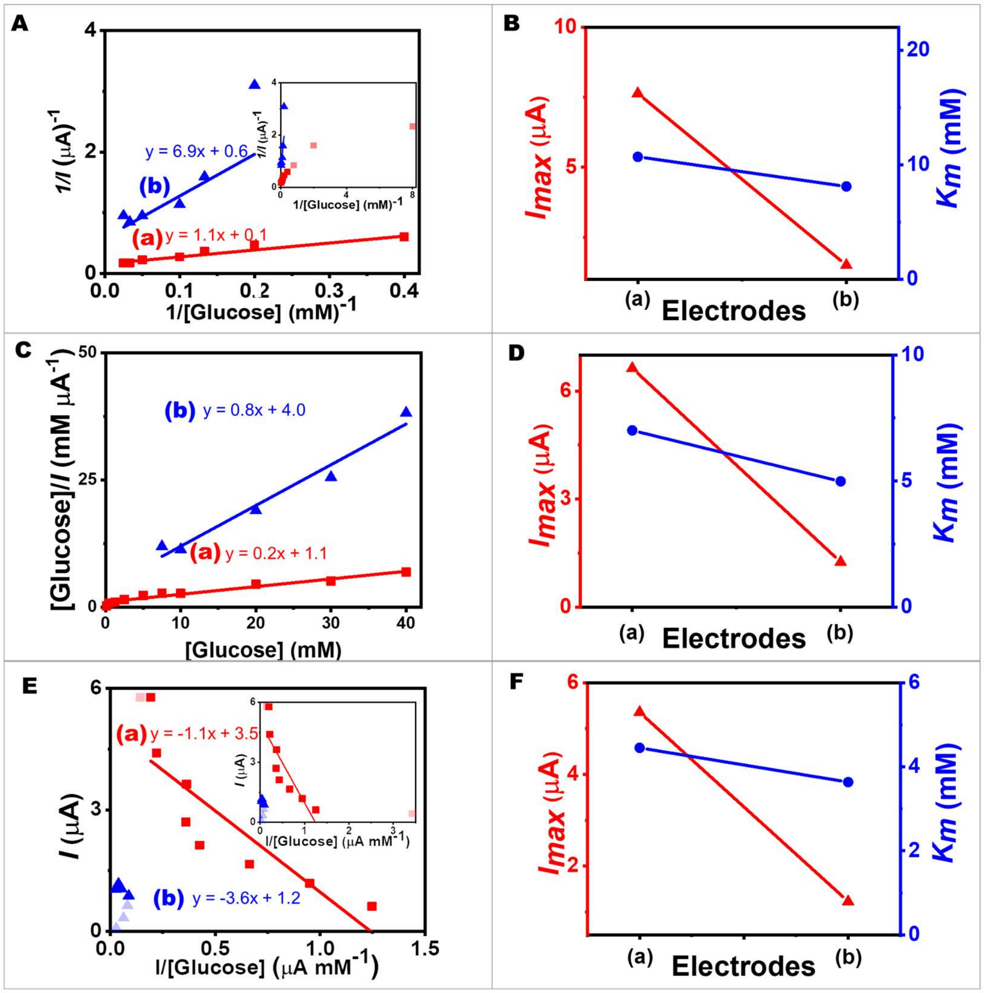

Figure S7. The plots used for estimating K_m and I_{max} referring to Figure S6. These screen-printed bioelectrodes (a–b) two different layout arrangement with the same total volume of 10 μL of 10%v/v egg white proteins, 10 μL of 10 mg mL^{-1} GOx (in 10%v/v egg white proteins), and 274 G-units of AuNPs with 750 nmol NQ. (A) The Lineweaver–Burk plot. (B) I_{max}, and K_m values estimated from the Lineweaver–Burk plot. (C) The Hanes-Woolf plot. (D) I_{max}, and K_m values estimated from the Hanes-Woolf plot. (E) The Eadie-Hofstee plot. (F) I_{max}, and K_m values estimated from the Eadie-Hofstee plot. Data points in light colors are excluded during fitting.

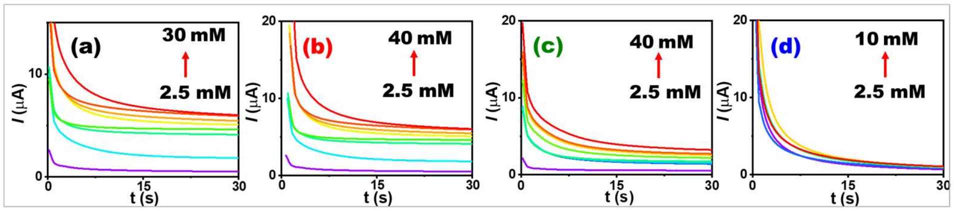

Figure S8. Amperograms of screen-printed bioelectrodes using different number of egg white proteins with an applied potential of 0.40 V vs. Ag/AgCl upon increasing the glucose concentrations. These electrodes (a–d) refer to Figure 4A. These screen-printed bioelectrodes with (a-d) 0, 10, 20, and 40 μL of 10%v/v egg white solution.

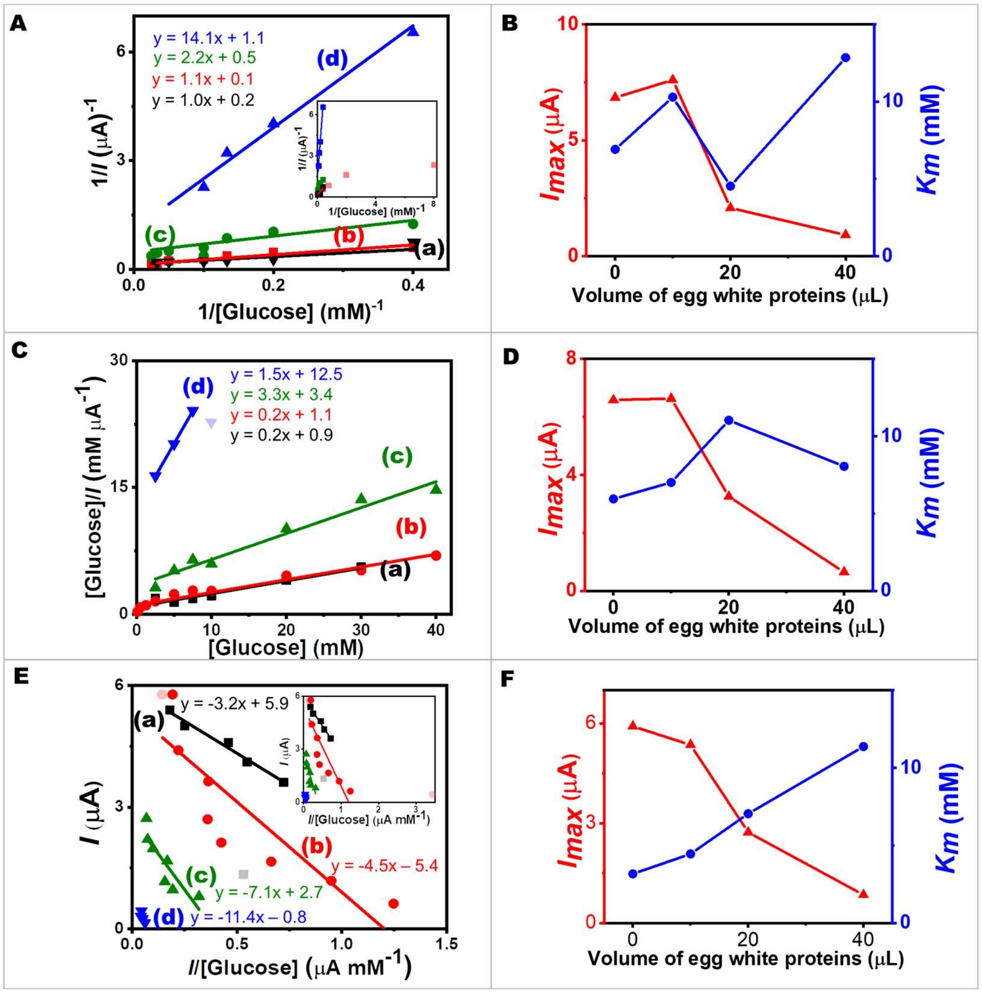

Figure S9. The plots used for estimating K_m and I_{max} referring to Figure S8. These screen-printed bioelectrodes with (a-d) 0, 10, 20, and 40 μL of 10%v/v egg white solution. (A) The Lineweaver–Burk plot. (B) I_{max}, and K_m values estimated from the Lineweaver–Burk plot. (C) The Hanes-Woolf plot. (D) I_{max}, and K_m values estimated from the Hanes-Woolf plot. (E) The Eadie-Hofstee plot. (F) I_{max}, and K_m values estimated from the Eadie-Hofstee plot. Data points in light colors are excluded during fitting.

Reference

1. Abeyrathne, E.D.N.S.; Lee, H.Y.; Ahn, D.U. Egg white proteins and their potential use in food processing or as nutraceutical and pharmaceutical agents—A review. *Poultry Science* **2013**, *92*, 3292-3299, doi:https://doi.org/10.3382/ps.2013-03391.
